# Corun: Concurrent Inference and Continuous Training at the Edge for Cost-Efficient AI-Based Mobile Image Sensing

**DOI:** 10.3390/s24165262

**Published:** 2024-08-14

**Authors:** Yu Liu, Anurag Andhare, Kyoung-Don Kang

**Affiliations:** Department of Computer Science, State University of New York at Binghamton, 4400 Vestal Parkway East, Binghamton, NY 13902, USA; yliu456@binghamton.edu (Y.L.); aandhar2@binghamton.edu (A.A.)

**Keywords:** AI-based image sensing, deep learning, concurrent inferences, retraining, edge computing

## Abstract

Intelligent mobile image sensing powered by deep learning analyzes images captured by cameras from mobile devices, such as smartphones or smartwatches. It supports numerous mobile applications, such as image classification, face recognition, and camera scene detection. Unfortunately, mobile devices often lack the resources necessary for deep learning, leading to increased inference latency and rapid battery consumption. Moreover, the inference accuracy may decline over time due to potential data drift. To address these issues, we introduce a new cost-efficient framework, called Corun, designed to simultaneously handle multiple inference queries and continual model retraining/fine-tuning of a pre-trained model on a single commodity GPU in an edge server to significantly improve the inference throughput, upholding the inference accuracy. The scheduling method of Corun undertakes offline profiling to find the maximum number of concurrent inferences that can be executed along with a retraining job on a single GPU without incurring an out-of-memory error or significantly increasing the latency. Our evaluation verifies the cost-effectiveness of Corun. The inference throughput provided by Corun scales with the number of concurrent inference queries. However, the latency of inference queries and the length of a retraining epoch increase at substantially lower rates. By concurrently processing multiple inference and retraining tasks on one GPU instead of using a separate GPU for each task, Corun could reduce the number of GPUs and cost required to deploy mobile image sensing applications based on deep learning at the edge.

## 1. Introduction

Intelligent mobile image sensing powered by DL (deep learning) analyzes images captured by cameras on mobile devices such as smartphones or smartwatches. In particular, CNNs (convolutional neural networks) support a broad spectrum of mobile image sensing applications, such as image classification, image search, object detection/recognition, face recognition, image denoising, depth estimation, and camera scene detection [1,2,3]. Moreover, state-of-the-art Vision Transformers [4,5,6,7] have demonstrated superior inference quality to CNNs (at the cost of higher complexity).

Unfortunately, mobile devices often lack the resources necessary for deep learning, leading to long inference latency and rapid battery consumption that can significantly degrade the quality of service perceived by users. Another challenge for mobile image sensing using DL is that inference accuracy can decline over time due to potential data drift, as small, specialized DL models for mobile image sensing are relatively less robust [8,9,10,11]. For example, lightweight DL models often suffer from low accuracy in poor lighting or severe weather, such as heavy rain or snow. Furthermore, a classification task may fail if it is given a category not trained for.

A viable approach to addressing these challenges is deploying edge servers in cellular networks (e.g., 5G or LTE) and Wi-Fi networks, to which mobile users can offload DL-based image-sensing workloads. Compared to image analysis in the cloud, edge computing has several advantages. First, communication latency and Internet bandwidth consumption can be significantly reduced. Second, a pre-trained DL model can be continuously (re)trained and updated on the edge server, using new sample images collected by devices to improve accuracy in the presence of data drift [8,9,10,11]. In addition, privacy concerns can be mitigated by processing data on an edge server located on-premises, instead of sending them to a public cloud.

Mobile edge computing, however, incurs the cost of deploying and managing edge servers. Most existing approaches have limitations in supporting mobile image sensing and handling potential data drift cost-efficiently:

- A common approach for low latency model serving involves exclusively using an entire GPU to process a single inference request at a time. However, solo inference suffers from low inference throughput. Moreover, it may increase the number of GPUs needed to support intelligent mobile image sensing via edge computing.
- Solitary training using the entire GPU is not cost-effective for AI-based mobile image sensing, either. No inference requests can be served if the GPU is dedicated to the training job, reducing inference throughput as a result.
- Recent work, such as [12,13,14], aims to support simultaneous inferences on the same GPU to improve inference throughput while managing latency. However, model retraining or updates to cope with data drift are not considered in these approaches.
- Time-sharing and fast job switching have been investigated to efficiently schedule training or inference jobs [15,16,17]. In [18], two training jobs are run on a single GPU via efficient memory management. However, most existing work, including [12,13,14,15,16,17,18], does not consider co-running continuous retraining for upholding accuracy alongside inference jobs on the same GPU in an edge server.

To address these challenges, we introduce a new scheduling framework called Corun. In contrast to most existing work, we designed Corun to simultaneously handle multiple DL inference queries (user requests) and continuous model retraining on a single commodity GPU. This approach, which uses a cost-effective edge server instead of expensive cloud GPUs, significantly improves inference throughput without substantially increasing latency, while maintaining accuracy. Unlike a solitary inference or training method, where one inference or retraining job uses the entire GPU until it completes, blocking other inference or retraining tasks, Corun enables multiple inference queries and a training task to share a single GPU. Thus, it could considerably reduce the number of GPUs and the cost required to deploy intelligent visual sensing applications and maintain accuracy by retraining a pre-trained model at the edge. (Hereafter, we use training and retraining interchangeably.)

To minimize the resource-demand for high-accuracy mobile image sensing based on DL, it is desirable if a relatively inexpensive commodity GPU, compared to its cloud counterparts, can host continuous training and inference jobs together. However, the feasibility of this approach is mostly unclear. Furthermore, GPUs use black-box, proprietary scheduling policies, the details of which are not disclosed to the public. To bridge the knowledge gap, in this paper, Corun effectively co-executes ephemeral inference jobs to serve image-sensing queries from users with an ongoing training job. A summary of our **goals is as** follows:

- G1: Significantly enhance the inference throughput.
- G2: Avoid a large, superlinear increase in the inference latency or epoch time in training, when more inferences are concurrently performed alongside a continuous training job on a shared GPU.
- G3: Ensure that inference/training jobs do not crash due to the excessive co-location of models in one GPU and resulting OOM (out-of-memory) errors.
- G4: Achieve G1–G3 with minimal complexity and overhead at runtime to efficiently serve multiple inference requests and a continuous training job simultaneously on the same GPU.

Our **key contributions** are summarized as follows:

- A pilot measurement study is performed to assess the feasibility of concurrent inferences and training on a commodity GPU.
- A reliable scheduling method for concurrent inferences and continuous training is designed to significantly enhance throughput, avoiding OOM errors and a large latency increase with negligible runtime overhead.
- For several well-established CNN models and two cutting-edge Transformer models for image dehazing [6,7], Corun enhances the inference throughput by up to 4.69× with moderate latency increases and little overhead at runtime, while avoiding OOM errors. Therefore, Corun meets G1, G2, G3, and G4.

The remainder of this paper is organized as follows. In Section 2, related work is discussed. In Section 3, we conduct a brief measurement study to analyze the feasibility of concurrent inferences and training on a commodity GPU. In Section 4, our proposed method for co-running inferences and continuous training, Corun, is described. The CNN and Transformer models, datasets, and implementations are described in Section 5. In Section 6, our evaluation results are described. In Section 2, related work is discussed. Section 7 discusses our limitations and future research issues. Finally, Section 8 concludes the paper.

## 2. Related Work

In this section, related work in different categories is discussed in comparison to Corun.

### 2.1. Solo Inference

Several model serving systems, including [19,20,21,22], have been developed to support low latency inference services using pre-trained models. Clipper [22] introduced caching, batching, and adaptive model selection techniques to reduce inference latency while improving throughput, accuracy, and robustness. INFaaS [23] enabled the selection and deployment of model variants, hardware, and scaling configurations to meet service-level objectives in terms of latency while improving throughput. However, different from Corun, these approaches process a single inference query using the entire GPU for short latency, leading to low inference throughput, as observed in Section 6. A comprehensive survey of techniques for efficient deep learning inference in edge devices, ranging from model compression to offloading, is given in [24]. However, the survey does not cover model retraining and concurrent inferences at the edge. Corun can serve more inference jobs concurrently if, for example, models can be compressed without a noticeable drop in accuracy. In addition, Corun supports model retraining essential for compressed models with less robustness [8,9,10,11] alongside concurrent inferences. Thus, it is complementary to other methods for efficient edge inference.

### 2.2. Advanced Deep Learning Models for Mobile Applications

Vision Transformers, such as [4,5], have recently advanced the quality of inference in vision tasks. This has introduced new opportunities for intelligent mobile applications and image sensing. For example, RIDCP [6] and DehazeDCT [7] are novel image dehazing models that play a central role in image enhancement and restoration. They leverage the Transformer and other advanced techniques, such as fast Fourier convolution (FCC), high-quality priors, and deformable convolution. Moreover, NightHazeFormer [25,26] support nighttime haze removal. In mobile applications, Corun can run such image-dehazing models on an edge server to enhance and restore images, reducing the battery consumption of mobile devices. In Section 6, we thoroughly evaluate Corun using RIDCP [6] and DehazeDCT [7] as well as various CNN models.

### 2.3. Concurrent Inferences

Several approaches have been proposed to process multiple inference tasks on one GPU. Perseus [12], for instance, executed ResNet50 and Inception-V3 together, achieving up to 12% cost reduction for model serving. Choi et al. [13] designed a new scheduler that explores 3D search space of different batch sizes, temporal sharing, and spatial sharing between two inference tasks in one GPU. At the DL operator level, Yu et al. [27] designed a search algorithm based on machine learning to run multiple inference tasks using the same input. Unlike [27], we do not assume that inference models analyze the same input because it is unrealistic to assume that users provide the same input for mobile image sensing. KRISP [28] spatially partitions a GPU at the kernel level to enhance GPU utilization. GSLICE [29] collocates multiple inference jobs that share a DL model. COLTI [30] attempts to extend GSLICE to support training in addition to serving inference requests using a common DL model; however, it is not clear how it avoids an OOM error or a large latency increase due to excessive co-location, unlike Corun. Moreover, Corun does not require concurrent inferences to use the same DL model, in contrast to GSLICE and COLTI. In [31,32], the concurrent running of inference tasks has been explored, enhancing the throughput of edge servers by 1.4× and 3.8×, respectively. However, continuous training for maintaining accuracy alongside concurrent inferences is not considered in these works.

### 2.4. Efficient Training

Gandiva [15] proposed a GPU time-slicing mechanism that allows job switching at the iteration boundaries in training, supporting second-scale suspend/resume. However, this method is too slow to support online inference queries with low latency requirements. Salus [16] supports fast job switching between iterations by retaining persistent memory of the preempted job, which is considerably smaller than the temporary memory used in a DL training job. SwitchFlow [17] treats a DL model as a computation graph to facilitate preemptive multitasking, scheduling subgraphs to prevent them from running simultaneously on a single GPU. When a low-priority subgraph is preempted, it can continue to run on a different device, such as the CPU or another GPU. Unfortunately, preemptive approaches, such as [15,16,17], have substantial overhead (up to 1 s and 110 ms in Gandiva and SwitchFlow, respectively). Zico [18] takes a non-preemptive approach that allows two concurrent training jobs to share a single GPU by reclaiming memory released by one model during the backward pass. Wavelet [33] supports efficient model and data parallel training based on a similar idea. Unlike these approaches, Corun devises a new non-preemptive approach that enables a continuous training job and several inference requests to run in parallel on an edge server with O(1) time complexity at runtime.

### 2.5. Continuous Learning at the Edge

Compressed DL models for efficient mobile/edge image sensing are relatively less robust and susceptible to accuracy drops due to data drift that can occur in a series of images or video frames. Continuous learning has emerged as a promising approach to maintain the accuracy of vision tasks at the edge, even in the presence of data drift [8,9,10,11]. To maintain accuracy, RECL [34] dynamically selects among the current CNN model, a retrained model, or one of the historic models that the edge server maintains in its model zoo. However, most existing strategies have not addressed the challenge of concurrently serving inference queries in a timely manner alongside an ongoing retraining job, as achieving it is hard without substantially increasing the resource demand. To shed light on this issue, Corun provides a new framework that accomplishes goals G1–G4 via concurrent CNN inferences and retraining based on effective offline profiling and runtime scheduling. Therefore, Corun is complementary to these works.

### 2.6. GPU Workload and Performance Predictions

Corun avoids OOM errors and long latency through offline profiling. In contrast, Gao et al. [35] estimated GPU memory consumption, Hu et al. [36] predicted the performance of memory-intensive GPU kernels, and Hu et al. [37] predicted deep learning workloads in GPU data centers. Despite their effectiveness, these prediction/estimation methods are subject to estimation or prediction errors, possibly leading to OOM errors or high latency. They could be used to reduce the search space and decrease the time for offline profiling in Corun. Thus, they are complementary to our work.

## 3. Motivation

For the work presented in this article, we first carried out a pilot study that involved deploying several CNN models in Table 1 and Table 2, which were used for various applications in mobile/edge environments [1,38,39]. Popular machine learning benchmarks, such as EdgeBench [2] and MLPerf [3], include CNNs. Furthermore, they have a wide range of model sizes and computational complexities (GFLOPs).

In this experiment, we conducted solo training and inference jobs, where each training or inference job used the entire GPU until it finished. We performed 1000 inferences and 10 training epochs for each model using the ImageNet dataset [40] to measure the average GPU utilization, power consumption, temperature, and frequency for an inference and training job, respectively.

**Table 1:** CNN training models (input image size: 224 × 224).

Models	Batch Size	Parameters	GFLOPs
MobileNetV3-Small [41]	128	2.5 M	0.1
ResNet50 [42]	16	25.6 M	4.1
EfficientNetV2-Large [43]	2	118.5 M	12.3

**Table 2:** CNN inference models (input image size: 224 × 224).

Models	Parameters	GFLOPs
MobileNetV3-Small [41]	2.5 M	0.1
ResNet50 [42]	25.6 M	4.1
EfficientNetV2-Large [43]	118.5 M	12.3
GoogleNet [44]	6.6 M	1.5
InceptionV3 [45]	23.8 M	2.9
DenseNet121 [46]	8.0 M	2.9

Table 3 and Table 4 present the GPU utilization, frequency, power consumption, and temperature for the training and inference of the CNN models in Table 1 and Table 2, respectively. As shown in the tables, the average utilization values are, at most, 35.2% and 38.2% for training and inference, respectively. Hence, a solo training or inference job underutilizes the tested commodity GPU (NVIDIA RTX 3080 Ti). The GPU power consumption is considerably lower than the 350W TDP (thermal design power) of the GPU. Furthermore, the GPU temperature is below 58 °C for a training and inference job, which is much lower than the 93 °C that triggers thermal throttling [47]. Thus, the GPU is thermally safe and is not subject to thermal throttling. Additionally, the GPU frequency ranges from 1755 to 1965 MHz while the average is below 1800 MHz, whereas our GPU supports an adjustable frequency that ranges between 210 and 2025 MHz with a step size of 15 MHz [48]. The results of this pilot study motivated our approach to achieving goals G1–G4 through concurrent inferences and training on a commodity GPU.

Furthermore, we find that *GPU memory is the most coveted bottleneck resource* because OOM errors occur when GPU memory becomes insufficient to accommodate several tasks simultaneously, even when other resources are available. To avoid devastating OOM errors, the underlying DL frameworks, such as PyTorch and TensorFlow, implement memory swapping. This involves temporarily transferring some memory blocks from the GPU to the host memory (RAM) using the NVIDIA UVM (unified virtual memory) mechanism. By freeing up GPU memory, this technique allows other jobs to continue executing, utilizing available resources. Swapped memory blocks can be brought back to the GPU when they are needed again. In this paper, however, we disable this feature because of the high, unpredictable latency and overhead of swapping. Instead, we spatially multiplex a GPU for robust sharing between concurrent inferences and continuous training, avoiding OOM errors or superlinear increases in inference/training latency without the need for high-end cloud GPUs.

## 4. Concurrent Training and Inferences

In this section, Corun is designed to achieve goals G1–G4. To achieve G1, Corun needs to find the maximal level of concurrency *K*, i.e., the number of concurrent inference jobs that can run alongside a continuous training job while maintaining accuracy. There are numerous options for concurrency levels and batch sizes that avoid OOM errors or a sharp increase in inference latency, meeting the requirements of G2 and G3; however, they may lead to different throughput levels related to attaining G1. It can be shown that finding an optimal solution using the finite resources available in a GPU is a knapsack problem, which is NP-Hard. Thus, it is very hard to attain G4 while achieving G1–G3.

To address this issue, we propose a cost-effective heuristic, Algorithm 1, based on offline profiling. To accomplish goals G1–G4, Algorithm 1 keeps increasing *K* offline until an OOM error occurs, or the latency grows superlinearly, i.e., faster than *K*. Thereby, Corun identifies the training and inference jobs that are compatible in that they can run concurrently to significantly improve inference throughput compared to solo inference, without incurring an OOM error or a rapid (superlinear) increase in latency for either inference or training.

**Figure 1:**
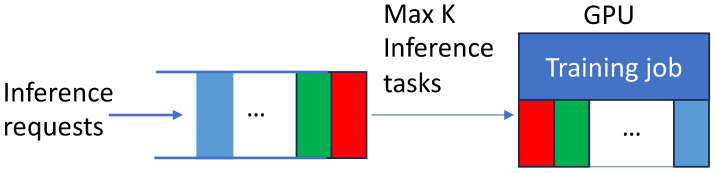
FCFS Scheduling and multiplexed dispatch for concurrent CNN training and inferences.

To efficiently schedule concurrent inference requests and continuous training at runtime, Corun extends FCFS (First Come, First Served) scheduling by spatial GPU multiplexing based on the stored results of offline profiling driven by Algorithm 1. As user inference requests arrive, Corun dispatches and executes the first *K* inference requests at the head of the FIFO queue with the ongoing training task, if there are *K* or more compatible inference queries in the queue. Otherwise, it executes all (less than *K*) inference queries in the FIFO queue and the training job, as illustrated in Figure 1. The components of Corun—except for this efficient runtime scheduling scheme—are processed offline. In this way, Corun intends to achieve G4 as well as G1–G3.
 **Algorithm 1:** Finding an effective concurrency level via offline profiling
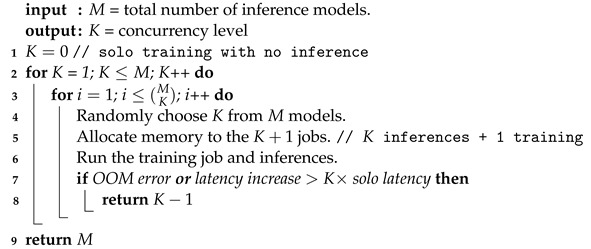


A more detailed discussion of our approach to finding an effective concurrency level (*K*) and batch size follows.

### 4.1. Finding a Maximal Feasible Concurrency Level

If the concurrency level is too low, precious GPU resources can be wasted, resulting in low throughput. On the other hand, if the concurrency level is too high, the inference latency or the time for an epoch or iteration of training may increase abruptly due to insufficient resources, such as the GPU memory and streaming multiprocessors (SMs). Even worse, training and inferences may crash due to an OOM error when the GPU memory becomes short. Unfortunately, predicting the GPU resource consumption of a DL model training/inference job before executing it is very hard for several reasons, including the following:

- The amount of GPU memory consumed by a CNN is not determined by the number of the model parameters only. For example, PyTorch, which is a popular DL framework, allocates GPU memory to in/out tensors, weight tensors, ephemeral tensors, and resident buffers to support CUDA contexts, memory alignment, and reservations [35], while supporting caching and dynamic memory management, such as memory defragmentation.
- DL frameworks, such as TensorFlow, PyTorch, and MXNet, hide the internal execution of a model from the high-level code written by developers, making it hard to monitor the GPU memory usage precisely. Moreover, analyzing low-level operators (e.g., convolution in CNNs) upon which a DL framework (e.g., TensorFlow, PyTorch, and MXNet) is built is difficult, because they are usually implemented using proprietary libraries (e.g., NVIDIA cuDNN and cuBLAS).
- Unlike feedforward inference, training requires holding temporary data (e.g., activations and gradients) until used during the backpropagation, further complicating GPU memory management.
- In general, GPU memory management and scheduling policies are proprietary and details are undisclosed.

To address these issues, Algorithm 1 determines a maximal number of concurrent inferences in addition to continuous training/updates of a specific CNN model. On line 1 of Algorithm 1, K=0, indicating solo training with no inference. On line 2, we increase *K* by 1. On line 4, we randomly choose *K* out of *M* models, where *M* is the total number of pre-trained models for model serving (i.e., inference services for DL-based mobile image sensing). On lines 5 and 6, we allocate GPU memory to *K* inference jobs in addition to the ongoing training jobs in proportion to their model sizes and concurrently execute them. On line 7, if an OOM error occurs or the inference latency or the epoch length of training grows faster than *K* times, Algorithm 1 returns K−1 on line 8. Otherwise, we repeat this up to MK times as specified on line 3. If lines 3–8 run successfully without being returned, we increment *K* by 1 and repeat the process as specified on line 2.

If there are *N* CNN models (one of which is trained at a time), Algorithm 1 is called for each CNN model *i*, where 1≤i≤N, and the result is stored in C[i], which represents the highest number of concurrent inferences that meet goals G1–G4 for the CNN model *i* to be retrained. If the CNN model *i* is being retrained at the runtime, Corun sets K=C[i] via a single table look-up with O(1) time complexity; therefore, Corun concurrently processes, at most, *K* inference queries alongside the retraining of the CNN *i* using the scheduling scheme depicted in Figure 1. Although Algorithm 1 finds *K* via an exhaustive search, it is efficient in practice: (1) *M* and *K* values (K≤M) are usually small due to the demanding resource requirements of DL models; (2) the search is immediately terminated and K−1 is returned if any of the three conditions on line 7 are violated; and (3) profiling is performed offline without delaying any inference or training job at runtime.

It is worth noting that Corun does not require control of the black-box GPU hardware scheduler nor modifications to the underlying hardware, drivers, or DL framework. Therefore, it is flexible and universally applicable, effectively supporting concurrent CNN training and inferences.

### 4.2. Batch Sizes for Inference and Training

The batch size is another important factor that affects throughput and GPU memory consumption. Generally, a large batch size for inference often improves throughput; however, it usually increases GPU memory consumption and latency. Moreover, it may affect the generalizability and GPU memory consumption of training. Thus, a proper configuration of batch sizes is desirable.

An inference task using a pre-trained CNN model only requires a single forward propagation pass through the model, which is a directed acyclic graph. Each node in the graph is an invocation of a mathematical operator (e.g., matrix multiplication), and each edge specifies the execution dependency. In this paper, we use the batch size of 1 for inference queries to support timely model serving; that is, an inference request for intelligent image sensing is not delayed for batching.

In training, each epoch consists of a specific number of iterations. Moreover, a single iteration consists of forward and backward propagation. The model parameters (i.e., weights) are updated during backpropagation based on the gradients, using an optimization algorithm, such as stochastic gradient descent or Adam. Thus, a single iteration makes one update of the model weights. If a training set has *N* samples (e.g., images) and a mini-batch consists of *B* samples, completing one epoch to learn the weights, using all *N* samples, takes $\frac{N}{B}$ iterations. Therefore, a small batch size, *B*, results in more iterations and more frequent weight updates in an epoch, but reduces the GPU resource usage in each iteration.

Nonetheless, a single epoch is completed when all *N* samples are used to learn parameters, regardless of the batch size, *B*. Moreover, the total number of epochs, *E*, is specified and fixed when a training job begins. Therefore, using a small batch size reduces resource consumption in each iteration, but does not affect the total training time in principle. More formally, when there are *B* samples in a batch, the O(B) computation is required using O(B) memory [49]. However, the uncertainty in the gradient is reduced by $O(\sqrt{B})$; that is, there are diminishing returns to increasing the batch size. Furthermore, in DL training, a small batch size can lead to faster convergence, better generalizability, and more stability [50,51,52]. Optimizing the batch size for DL training is still an open problem [49,50,51,52]. In this paper, we use relatively small batch sizes for continuous training in Table 1 to strike a balance between the conflicting requirements for more concurrent inferences and more samples in a batch for reliable training, considering diminishing returns to larger batch sizes with higher resource demands per iteration.

## 5. CNN Models, Transformer Models, Datasets, Performance Metrics, and Implementation

In this section, CNN models, Transformer models, datasets, evaluation metrics, and our implementation are described.

### 5.1. Deep Learning Models for Retraining and Inference

- Retraining Models: As shown in Table 1, to generate retraining/fine-tuning workloads, we selected MobileNetV3-Small, ResNet50, and EfficientNetV2-Large with relatively small, medium, and large sizes, and GFLOPs to analyze the impact of concurrent training on inference performance and vice versa. MobileNet has been extensively studied for its applications in mobile and edge computing environments [53,54]. Its effectiveness in managing AI tasks has made it a key component in state-of-the-art networks, with significant use in the SSD (single-shot detector) and YOLO (you only look once) series for object detection [55,56,57]. Residual connections featured in ResNet50 are still utilized in Vision Transformers [4,5]. In addition, EfficientNetV2 has a faster training speed and better parameter efficiency than previous models. In this paper, an edge server trains zero or one model in Table 1 and simultaneously serves incoming inference requests.
- Inference models: To analyze the performance of concurrent inferences in the absence and presence of a training job, we use six popular CNN models and two state-of-the-art Transformer models for image dehazing, i.e., RIDCP [6] and DehazeDCT [7].

### 5.2. Datasets

To analyze inference performance in terms of throughput (QPS) and latency, we use CNN models pre-trained using the ImageNet dataset [58]. Furthermore, to evaluate the inference performance of the pre-trained RIDCP [6] and DehazeDCT [7] models, we use the NH-HAZE [59] dataset for image dehazing. For continuous (re)training of the models in Table 1, we utilize the Mini-ImageNet dataset [60], comprising 50,000 training images and 10,000 testing images evenly distributed among 100 classes. Typically, a lightweight CNN model is specialized for a subset of categories to enable efficient inference at the edge [8,9,10,11]. To maintain the accuracy of edge inference, the specialized model is then periodically retrained on an edge server to handle potential data drift. In each update phase, a relatively small number of iterations are performed using recent sample images [8,9,10,11]. In particular, we train one of the models in Table 1 for 10 epochs to emulate periodic updates in each set of experiments, where concurrent CNN inference queries are served alongside an ongoing job for retraining a CNN model. In this regard, we use Mini-ImageNet, which is significantly smaller than ImageNet, to emulate CNN model updates on an edge server using fresh sample images to maintain inference accuracy. However, in each epoch, we stress-test Corun by training a CNN model using the entire 50,000 images in the training set of Mini-ImageNet, which is significantly larger than the number of samples used for a continual update of a CNN model in an edge server [8,9,10,11].

### 5.3. Performance Metrics

- Inference throughput: We measure the queries per second (QPS), which measure the number of inference requests processed per second.
- Inference latency: The latency for model serving is one of the key metrics to measure the user-perceived quality of service. Specifically, we measure the average and 95-P tail latency of inferences to analyze their ascending patterns when more CNN inference queries are run simultaneously.
- Epoch length: We measure the average time for each model in Table 1 to finish one training epoch with respect to different numbers of co-running inference tasks.

In this paper, accuracy is not a performance metric, as we use pre-trained CNN models for inferences. Moreover, we assume that continuous training maintains accuracy even in the presence of data drift [8,9,10,11]. Given that, we analyze the impact of continuous training on inference throughput and latency, while evaluating the impact of concurrent inferences on the training epoch time.

### 5.4. Implementation

To support concurrent executions, we exploit MPS (multi-process service) [61]. In particular, we use MPS to create multiple CUDA streams and allocate GPU memory to them in proportion to their model sizes.

For performance evaluation using CNNs, we use a PC to mimic a cost-effective edge server. It comprises commodity hardware components: an Intel Core i7-7820X CPU, 64 GB of RAM, and an NVIDIA GeForce RTX 3080Ti GPU with 12 GB of GDDR6X memory.

As Transformers require more GPU memory, we utilize a different PC that has an NVIDIA GeForce RTX 4090 GPU with 24 GB of memory. It is used for all experiments that include one or more inference jobs using RIDCP [6] or DehazeDCT [7]. The remaining hardware configuration is similar. On the machine with an NVIDIA GeForce RTX 3080Ti GPU with 12 GB of memory, only up to two concurrent inferences for image dehazing can be performed in addition to a retraining job. In this way, we also demonstrate the general applicability of Corun to different model architectures and GPUs. The implementation and evaluation of deep learning models are carried out using Python 3.9 and PyTorch 1.13. The machine with the NVIDIA GeForce RTX 3080Ti GPU runs Ubuntu 18.04.6 LTS, while Ubuntu 22.04.4 LTS is installed on the other workstation. The GPUs use NVIDIA’s GPU Boost technology, which automatically adjusts the clock speed based on various factors, such as power consumption, temperature, and workload.

**Figure 2:**
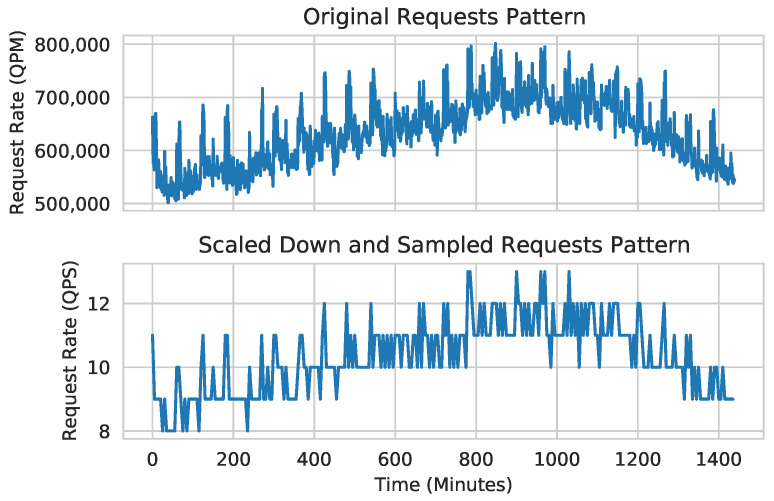
Original and scaled-down request patterns of Microsoft Azure FaaS function invocation traces.

To evaluate the throughput and latency of the concurrent model training and inferences using realistic request arrival patterns for online services, we adopt Microsoft Azure FaaS (function as a service) traces [62]. These traces consist of invocations per minute for various Azure functions collected over a 24-h period for 14 days. For performance evaluation, we adapt the traces from day 01. As we aim to generate realistic traffic of requests for mobile image sensing on a single edge server instead of a cloud with enormous resources, we adjust the request arrival rate to a range between 8 and 52 queries/second. Subsequently, we produce a new trace by sampling the request rate at every 5th minute out of 1440 min of a day. Figure 2 visually depicts the Azure request arrival pattern [62] and our request arrival pattern. As shown in the figure, the request arrival patterns remain consistent despite the adjustments.

Our offline profiling results using Algorithm 1 show that the specific GPU used in this paper can support up to four inference tasks and one training job, which continuously trains one of the models in Table 1, at the same time. Otherwise, an OOM error or superlinear latency increase occurs. However, Corun is not tied to a specific GPU. Instead, it is compatible with most modern NVIDIA GPUs with post-Volta architecture that supports MPS. It can concurrently serve additional inference queries for mobile image sensing in a more powerful GPU (or fewer concurrent queries in a less potent GPU) by applying Algorithm 1.

For concurrent CNN inferences, we use 162 unique combinations: 162 = 81 + 82 + 83 + 84 to run 1, 2, 3, and 4 concurrent inference models, respectively. In total, we use 648=162+3×162 combinations to concurrently run inferences alongside no training job and 1 out of 3 training jobs in Table 1, respectively. Upon a request’s arrival, 1–4 inference jobs are triggered simultaneously according to the level of concurrency employed in the corresponding combination. In addition, we use 32 combinations for concurrent dehazing inferences. Thus, a total of 680 combinations are used in our evaluation.

## 6. Evaluation

The performance of Corun is compared to solo inference and training using the entire GPU, which is a common practice for model serving and training.

### 6.1. Inference Throughput and Latency

Table 5 shows the results of the inference performance evaluation. In the table, the first column shows the model being trained. The second column shows the number of co-running inference tasks. In the other columns, the average latency, 95-P latency, and QPS values for a specific inference model are normalized to those of the model’s solo inference using the entire GPU exclusively, without any other concurrent inference or training.

The results in Table 5 are summarized as follows:

- CNN inference throughput and latency: The inference throughput, i.e., QPS, scales linearly, by up to 4.69× for EfficientNet, as the concurrency level, *K*, increases from 1 to 4. However, the normalized average inference latency increases at significantly slower rates, by up to 1.13× (DenseNet121). The 95-P latency also increases sublinearly, by at most 1.53× (Inception) due to the effective scheduling of Corun. The average and 95-P latency for EfficientNetV2, which is the slowest among the tested CNN models, are 37 ms and 50 ms, respectively.
- Transformer inference throughput and latency: As *K* increases from 1 to 4, QPS rates increase by up to 3.7× and 3.15× for RIDCP and DehazeDCT, respectively. However, as *K* increases from 1 to 4, the normalized average inference latency increases marginally at significantly slower rates. Specifically, it increases by up to 1.05× and 1.28× for RIDCP and DehazeDCT, respectively. Moreover, the 95-P latency of RIDCP and DehazeDCT increases by up to 1.04× and 1.42×, respectively. For RIDCP, the average latency increases from 157.54 to 165.41 ms, and the 95-P latency increases from 181.39 to 188.9 ms. For DehazeDCT, the average latency increases from 3.92 to 5.01 s and the 95-P latency increases from 4.5 to 6.4 s. In general, the Transformer models are significantly slower than the CNN models. Also, the increase rates in QPS for higher K values are lower than those of the CNN models due to their complexities.

In all experiments, Corun avoided OOM errors by design (Algorithm 1). In some cases, the latency in Table 5 decreases slightly as *K* increases. We observed that the hardware scheduler increases the clock frequency to cope with higher workloads in these cases.

### 6.2. Training Epoch Time

**Figure 3:**
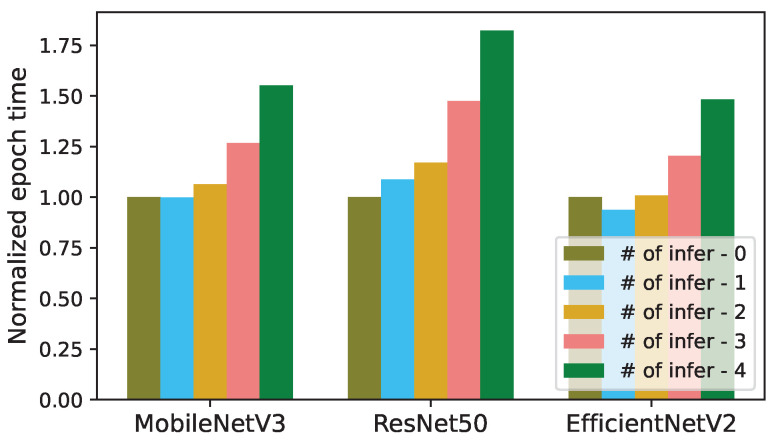
Normalized epoch times of training in the presence of concurrent CNN inferences (# of infer: number of concurrent inferences).

**Figure 4:**
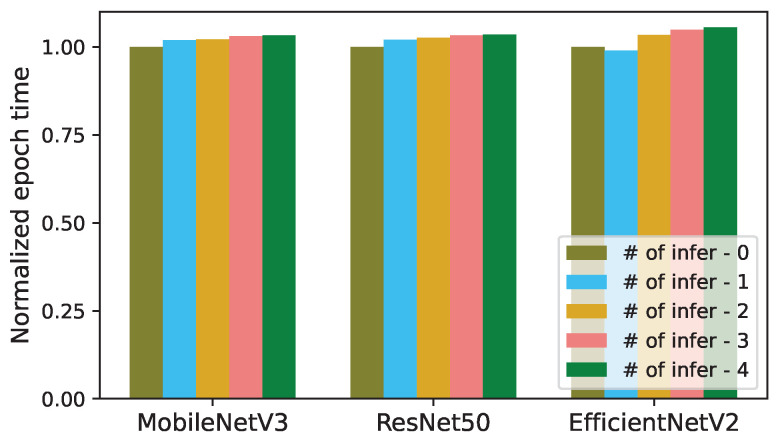
Normalized epoch times of training in the presence of concurrent Transformer inferences (# of infer: number of concurrent inferences).

We have analyzed the impact of concurrent inferences on the length of an epoch for training the models in Table 1. In Figure 3 and Figure 4, the epoch length of each trained model (MobileNetV3, ResNet50, and EfficientNetV2) is normalized to the epoch time of its solo training without any concurrent inference.

Similar to the inference latency, the epoch times in Figure 3 and Figure 4 also increase sublinearly with respect to *K*. The epoch lengths of MobileNetV3, ResNet50, and EfficientNetV2 increase by up to 1.58×, 1.82×, and 1.55×, respectively, when each one is run with 4 concurrent CNN inference tasks, as illustrated in Figure 3. When *K* increases from 1 to 4, the actual epoch times of MobileNetV3, ResNet50, and EfficientNetV2 increase from 236.6 to 367.5 s, 226.1 to 412.1 s, and 3319.4 to 5253.7 s, respectively. (In Figure 3 and Figure 4, the epoch length for EfficientNetV2 decreases as *K* increases from 0 to 1. This is because, in this case, the average GPU clock frequency increases from 1755 to 1944 MHz by the GPU hardware scheduler.).

Figure 4 illustrates that the epoch lengths of MobileNetV3, ResNet50, and EfficientNetV2 increase by up to 1.035×, 1.033×, and 1.056×, respectively, when each training job is run with 4 concurrent Transformer inferences for dehazing. When *K* increases from 1 to 4, the actual epoch times of MobileNetV3, ResNet50, and EfficientNetV2 increase from 218.85 to 226.69 s, 242.02 to 250.1 s, and 1967.71 to 2078.23 s, respectively. Thus, the impact of concurrent Transformer inferences on the training epoch time is insignificant. This is potentially because the Transformer experiments are undertaken in the NVIDIA Geforce RTX 4090 GPU, which has additional memory and other resources compared to the NVIDIA Geforce RTX 3080 GPU used for the CNN experiments.

These results are acceptable, considering that we stress-test Corun by analyzing the 50,000 images in the training set of Mini-ImageNet per epoch during continuous training, a volume significantly larger than the number of sample images typically used for retraining CNNs on an edge server [8,9,10,11]. Moreover, ResNet50 and EfficientNetV2 are larger than MobileNetV3 by one and two orders of magnitude, respectively (Table 1).

In general, our findings verify that Corun achieves goals G1–G4 cost-effectively. Corun is able to scale the QPS linearly with the number of co-running inference queries while avoiding OOM errors and preventing a superlinear increase in latency. Moreover, its runtime complexity is O(1).

## 7. Discussion

The work presented in this article has its limitations and future research issues, including the following:

- Flexible resource management: In this paper, we disabled UVM for low latency inferences. However, if the impact of swapping between the GPU and host memory on latency can be significantly reduced using, for example, prefetching [63], more training/inference tasks could run together to further enhance throughput. As another example, resources released by a training job during the backpropagation can be dynamically reclaimed to serve more inference requests. A thorough investigation is reserved for future work.
- Adaptive retraining based on estimated data drift: In this paper, we considered an extreme scenario where there is a persistent demand for retraining a CNN model to evaluate Corun in harsh conditions. Instead, retraining can be triggered only when considerable data drift is detected or predicted, processing more concurrent inference queries during periods where retraining is not needed. For example, in [34,64], a camera sends sample images to the edge server for model retraining upon a noticeable change of labels in semantic segmentation and object detection, respectively. In [11], retraining is triggered if adversarial autoencoders detect significant divergence in feature maps. Related research issues include developing more effective methods for predicting potential data drift and efficiently scheduling retraining and inference workloads accordingly.
- On-device inference and offloading: In this paper, we assume all inference queries are offloaded to an edge server. However, inference using a lightweight CNN, such as MobileNetV3, can be processed on a mobile device, where the model is continually updated by the edge server utilizing new samples provided by devices. In such scenarios, an edge server can support more users; a less powerful edge server can be employed to reduce costs. Optimizing data drift detection, sample data collection, model updates and downloads, as well as the consumption of computational resources and communication bandwidth for timely, high-accuracy inference, emerges as a critical area for future research. Furthermore, a hybrid approach, which dynamically balances inference workloads between devices and the edge server, considering the available communication bandwidth and the current status of devices and the edge server, can be explored.
- Autoscaling: Nexus [65], FA2 [66], and Cocktail [67] support autoscaling for model serving. Sia [68] introduces an adaptive DL-cluster scheduling scheme that is aware of GPU heterogeneity. DeepBoot [69] uses idle GPUs in the inference cluster for training tasks. Corun could be combined with these approaches. For example, it can be extended to support elastic scaling, enabling the utilization of additional GPUs, if necessary, to deal with flash inference requests.
- Vision Transformers: Novel Vision Transformers (ViTs), such as [4,5], have improved the quality of computer vision tasks. However, ViTs also increase computational and memory demands due to their self-attention mechanisms with quadratic complexity. This is one of the main reasons why CNNs are still popular for edge computing with relatively scarce resources [1,38,39]. For instance, in Section 6, the average and 95-P latency of DehazeDCT [7] are 3.92 and 4.5 s, respectively, even when the model is executed using the entire GPU with no other concurrent inference/retraining task. This is another limitation of our work presented in this manuscript, as short latency times (e.g., less than 1 s) are desirable for mobile applications. Further enhancements to Corun, such as improved scheduling and resource management, along with optimizations like compression and pruning of state-of-the-art models such as RIDCP [6], DehazeDCT [7], and NightHazeFormer [25], used to improve intelligent mobile applications, could be an exciting area for future research.
- Fault isolation: High-end NVIDIA GPUs, such as A100 and H100, support MIG (multi-instance GPU) [70], which enables hardware-level partitioning of one GPU into multiple instances with strong isolation. In this paper, however, Corun utilizes MPS available in most modern GPUs instead of MIG to reduce the cost of deploying image sensing models at the edge. Consequently, Corun has a limitation in terms of fault isolation. If an inference or a fine-tuning task fails, it may affect the other tasks concurrently running on the same GPU, potentially leading to a cascading failure. Extending Corun to support fault isolation without requiring expensive cloud GPUs capable of hardware-level isolation is a challenge. A possible direction is to leverage virtualization techniques in an edge server or a mini cluster of edge servers with several commodity GPUs while exploring effective scheduling algorithms for fault-tolerant inference and retraining. For example, inference or retraining tasks can be built as virtualized containers (e.g., [71]). Moreover, the fault tolerance features provided by the orchestrating framework (e.g., [72]) can be extended to support fault tolerance for concurrent inference and retraining tasks. A thorough investigation is reserved for future work.

Our study explores largely untapped opportunities for improving throughput without significant increases in latency through concurrent model retraining and inference. Encouraged by promising results, we plan to thoroughly investigate related research issues in the future, including those outlined in this section.

## 8. Conclusions

Intelligent mobile image sensing facilitated by deep learning analyzes images captured by cameras of mobile devices. It supports many mobile applications, such as image classification, face recognition, depth estimation, and camera scene detection. Mobile devices, however, often lack the resources necessary for deep learning, leading to long inference latency and quick battery consumption. Furthermore, the inference accuracy may drop over time due to possible data drift. To shed light on these issues, we introduce a new framework, called Corun, designed to simultaneously process multiple inference queries and continuous training on a single consumer GPU in an edge server to significantly improve the inference throughput and maintain accuracy, respectively. Our evaluation results demonstrate that Corun is cost-efficient; that is, the inference throughput scales linearly with the number of inference tasks that run concurrently with a continuous training job. However, the inference latency and epoch time of model training grow at substantially slower rates. Furthermore, Corun avoids expensive swapping or catastrophic out-of-memory errors. Thus, our efforts reveal fresh possibilities for enhancing the efficiency of model serving and retraining for mobile image sensing based on deep learning at the edge, potentially using fewer GPUs compared to solitary inference or retraining methods that require exclusive usage of a GPU for only one inference or retraining task at a time. In the future, we will investigate related leading-edge research issues, including those discussed in Section 7.

## Figures and Tables

**Table 3:** GPU utilization, frequency (kHz), power consumption (W), and temperature (°C) for solo training.

Model	Util.	Freq.	Power	Temp.
MobileNetV3-Small	4.6%	1755.0	119.4	50.4
ResNet50	29.2%	1861.9	190.6	57.5
EfficientNetV2-Large	35.2%	1756.0	146.6	55.1

**Table 4:** GPU utilization, frequency (kHz), power consumption (W), and temperature (°C) for solo inference.

Model	Util.	Freq.	Power	Temp.
EfficientNetV2-Large	18.4%	1755.0	128.6	49.0
MobileNetV3-Small	8.6%	1755.0	116.0	48.0
DenseNet121	20.9%	1755.0	126.6	48.8
ResNet50	20.4%	1755.0	132.5	49.2
InceptionV3	21.3%	1755.0	123.2	48.7
GoogleNet	14.3%	1759.3	121.4	49.0

**Table 5:** Normalized average latency, 95-P latency, and QPS. Data are normalized to the values of the inference of a single model without training. (*K* = the number of co-running inference models).

Training Model	*K*	MobileNet			ResNet50			GoogleNet			Inception			DenseNet121			EfficientNet			RIDCP			DehazeDCT		
		Avg	95-P	QPS	Avg	95-P	QPS	Avg	95-P	QPS	Avg	95-P	QPS	Avg	95-P	QPS	Avg	95-P	QPS	Avg	95-P	QPS	Avg	95-P	QPS
No Training	1	1	1	1	1	1	1	1	1	1	1	1	1	1	1	1	1	1	1	1	1	1	1	1	1
	2	0.97	0.97	1.97	0.99	1.07	2	1	1.09	2.01	1.01	1.12	2.05	1.01	1.11	2.09	1.03	1.06	2.25	0.99	1.01	1.92	1.03	1.18	1.95
	3	0.99	1.04	2.92	1.01	1.18	2.98	1.01	1.2	2.99	1.03	1.25	3.08	1.04	1.3	3.15	1.05	1.18	3.49	1.01	0.99	2.80	1.11	1.3	2.73
	4	0.98	1.17	3.83	1.02	1.4	3.93	1.02	1.36	3.94	1.06	1.47	4.07	1.09	1.45	4.18	1.08	1.27	4.69	1.05	0.99	3.7	1.27	1.4	3.12
MobileNet V3-Small	1	0.97	0.91	1.02	1.02	1.03	1.02	1	1.03	1.03	1.01	1.05	1.02	1.04	1.19	1.03	1.02	1.11	1.01	0.99	1.01	1.01	1.02	1.03	1.02
	2	1	1.01	2	1.02	1.12	2.03	1.01	1.16	2.04	1.03	1.18	2.08	1.03	1.23	2.12	1.03	1.16	2.28	1.01	1.02	1.92	1.03	1.19	1.96
	3	0.99	1.09	2.94	1.04	1.28	3	1.02	1.25	3.01	1.05	1.31	3.11	1.06	1.33	3.18	1.06	1.25	3.52	1.02	1.03	2.82	1.12	1.31	2.74
	4	1	1.21	3.83	1.04	1.44	3.92	1.05	1.41	3.94	1.09	1.52	4.06	1.1	1.49	4.17	1.1	1.33	4.68	1.03	1.04	3.69	1.27	1.41	3.15
ResNet50	1	1	0.97	1.01	1.01	1.09	1.01	1.02	1.06	1.02	1.05	1.19	1.02	1.06	1.2	1.03	1.03	1.12	1.01	1	1.01	1.01	1.01	1.02	1.02
	2	1.01	1.05	1.99	1.03	1.14	2.02	1.01	1.12	2.03	1.05	1.21	2.07	1.05	1.26	2.11	1.06	1.19	2.27	1.03	1.02	1.91	1.04	1.21	1.96
	3	1	1.11	2.94	1.04	1.27	2.99	1.04	1.26	3.01	1.08	1.32	3.1	1.07	1.33	3.16	1.08	1.26	3.51	1.02	1.03	2.83	1.12	1.32	2.73
	4	1.01	1.26	3.84	1.05	1.46	3.93	1.06	1.46	3.95	1.11	1.53	4.08	1.13	1.5	4.18	1.12	1.35	4.69	1.05	1.04	3.7	1.28	1.42	3.12
EfficientNet V2-Large	1	0.99	0.94	1	1	1.03	1.03	1.06	1.26	1.01	1.03	1.21	1.01	1.05	1.33	1.01	1.03	1.16	1	1	1.01	1.01	1.01	1.03	1.01
	2	0.97	0.98	1.98	1.02	1.14	2.01	1	1.1	2.02	1.03	1.21	2.06	1.04	1.31	2.1	1.04	1.19	2.26	1.01	1.03	1.91	1.04	1.20	1.95
	3	0.97	1.07	2.93	1.02	1.23	2.99	1.02	1.23	3.01	1.05	1.26	3.09	1.06	1.34	3.16	1.07	1.31	3.5	1.01	1.03	2.82	1.12	1.31	2.73
	4	1	1.21	3.84	1.03	1.4	3.93	1.03	1.41	3.95	1.09	1.46	4.08	1.11	1.46	4.19	1.11	1.39	4.69	1.03	1.04	3.69	1.27	1.41	3.13

## Data Availability

Our source code is available at https://github.com/Real-Time-Lab/Measuring-the-Throughput-and-Tail-Latency-of-Concurrent-Model-Training-and-Inferences (accessed on 3 June 2024).
